# Handlungsempfehlungen für SARS-CoV-2-Testkonzepte für asymptomatische Beschäftigte im Gesundheitswesen

**DOI:** 10.1007/s40664-023-00496-y

**Published:** 2023-03-01

**Authors:** Susanne H. Liebe, Anna Walendi, Lukas Brethfeld

**Affiliations:** Arbeits- und Gesundheitsschutz der Hochschulmedizin Dresden, Universitätsklinikum Carl Gustav Carus, Fetscherstr. 74, 01307 Dresden, Deutschland

**Keywords:** HCWs, Gesundheitsschutz, Infektionskontrolle, Coronavirus, Covid-19, HCWs, Occupational health, Infection control, Coronavirus, Covid-19

## Abstract

**Hintergrund und Zielsetzung:**

Bei der Umsetzung von SARS-CoV‑2-Testkonzepten in Einrichtungen des Gesundheitswesens kommen ranggleiche Gesetze und Verordnungen zur Anwendung. Vor dem Hintergrund erlebter Hindernisse bei einer adäquaten Umsetzung gesetzlicher Vorgaben in regelkonforme und rechtssichere Prozesse auf betrieblicher Ebene war das Ziel der vorliegenden Arbeit, dafür konkrete Handlungsempfehlungen zu entwickeln.

**Methodik:**

In einer Fokusgruppe mit Vertreter*innen aus Behörden, Fachschaften und Interessenvertretungen wurden auf Basis von Leitfragen aus zuvor identifizierten Handlungsfeldern in einem holistischen Ansatz kritische Aspekte der Umsetzung diskutiert. Die transkribierten Inhalte wurden anhand deduktiv-induktiver Kategorienbildung qualitativ analysiert.

**Ergebnisse:**

Alle Diskussionsinhalte konnten den identifizierten Kategorien *Gesetzliche Hintergründe von, Anforderungen an und Zielrichtungen für Testkonzepte in Gesundheitseinrichtungen, Zuständigkeiten für Umsetzung in betrieblichen Entscheidungsketten *sowie *Implementierung von SARS-CoV-2-Testkonzepten/Testprozessen* zugeordnet werden.

**Implikationen:**

Die Umsetzung gesetzlicher Vorgaben in regelkonforme und rechtssichere SARS-CoV-2-Testkonzepte in Einrichtungen des Gesundheitswesens setzt bereits sowohl die Einbeziehung von Ministerien, Fachschaftsvertretungen, Berufsverbänden, Arbeitgeber(AG)- und Arbeitnehmer*innenvertretungen, Datenschutzexpert*innen, Vertretungen möglicher Kostenträger als auch eine integrative und vollzugstaugliche Formulierung von Gesetzen und Verordnungen voraus. Die Definition von Zielrichtungen für Testkonzepte ist maßgeblich für nachfolgend im Betrieb etablierte Prozessabläufe, bei denen Aspekte des Beschäftigtendatenschutzes genauso berücksichtigt werden müssen wie die Bereitstellung zusätzlicher personeller Ressourcen für die Bewältigung der Aufgaben. Zudem muss es in Zukunft ein zentrales Anliegen der Betriebe sein, IT-Schnittstellenlösungen für eine datenschutzkonforme Informationsweitergabe zu Beschäftigten zu finden.

## Abstract (en)

**Background and objective:**

When implementing SARS-CoV‑2 testing concepts in healthcare facilities, different laws and regulations of equal rank apply. In the light of experienced impediments in translating legal requirements appropriately into legally secure concepts on an operational level, the objective of this paper was to develop corresponding specific recommendations for action.

**Methods:**

On the basis of guiding questions on previously identified fields of action, a focus group consisting of representatives of administration, different medical disciplines, and special interest groups discussed critical aspects of implementation using a holistic approach. The transcribed contents were analyzed through inductive development and deductive application of categories.

**Results:**

All contents of discussion could be matched with the identified categories legal backgrounds, requirements and objectives of testing concepts in healthcare facilities, responsibilities for implementing in operational decision-making chains, and implementing SARS-CoV‑2 testing concepts.

**Implications:**

The correct implementation of the legal requirements into legally compliant SARS-CoV‑2 testing concepts in healthcare facilities previously required the involvement of ministries, representatives of different medical disciplines and professional associations, employer and employee representatives and data privacy experts as well as representatives of possible cost bearers. In addition, an integrative and enforceable composition of laws and regulations is necessary. Defining objectives for testing concepts is significant for the following operational process flows that need to consider aspects of employee data privacy as well as providing additional personnel for fulfilling the tasks. Also, in future one central issue of healthcare facilities concerns the finding of solutions for IT interfaces for information transfer to employees in accordance with data privacy.

Im Januar 2020 hat die Corona-Pandemie Deutschland erreicht. Mit ihrer ersten Welle hat sie spätestens im März/April des Jahres die medizinischen Versorgungssysteme das erste Mal an die Grenzen ihrer Leistungsfähigkeit gebracht [1]. Zu diesem Zeitpunkt waren Bundes- und Landespolitik sowie örtliche Behörden, aber auch betriebliche Entscheider*innen gefordert, Hygiene- und Schutzkonzepte zu entwickeln, die einer Weiterverbreitung der SARS-CoV-2-Infektionen in der Gesellschaft entgegenwirken.

Circa 5 Mio. Beschäftigte im Gesundheitswesen („health care workers“, HCWs), übernehmen in der aktuellen COVID-19-Pandemie eine maßgebliche und verantwortungsvolle Schlüsselfunktion für eine kontinuierliche und qualitätsgesicherte Versorgung von Patient*innen [2].

Für die Sicherstellung der medizinischen Versorgung der Bevölkerung ist die Unterbrechung von Infektionsketten in diesem Setting zwingend notwendig. Auch nach der Einführung der *einrichtungsbezogenen Impfpflicht* müssen weiterhin effektive Strategien für Beschäftigtentestungen umgesetzt werden. Grassly et al. [3] haben den Einfluss verschiedener Test- und Isolationsstrategien auf die Transmission von SARS-CoV‑2 überprüft. Den Ergebnissen zufolge reduzieren wöchentliche RT-PCR-Testungen (Reverse-Transkriptase-Polymerase-Kettenreaktion, kurz PCR) inklusive asymptomatischer HCWs und asymptomatischer Patient*innen der Hochrisikogruppen, die Infektionstransmission um 23 %. Evans et al. [4] berechneten mittels SEIR-Modells die Reduzierung von Infektionen um 37 % unter HCWs durch regelmäßige PCR-Tests.

Bereits im September 2020 wurde durch die Deutsche Interdisziplinäre Vereinigung für Intensiv- und Notfallmedizin (DIVI) eine diesbezügliche S1-Leitlinie „SARS-CoV-2-Infektion bei Mitarbeiterinnen und Mitarbeitern im Gesundheitswesen – Bedeutung der RT-PCR-Testung“ veröffentlicht. Mit der Leitlinie wurden konkrete Handlungsempfehlungen zur Durchführung von RT-PCR-Testungen auf SARS-CoV-2-Infektionen nicht nur zum Schutz des Personals, sondern auch zum Schutz der Betreuten verabschiedet. Testungen sollten – unabhängig von bestehenden Symptomen – in Abhängigkeit vom konkreten Tätigkeitsbereich und der damit verbundenen konkreten Infektionsgefährdung durchgeführt werden. Weitere Empfehlungen sehen vor, die lokale bzw. regionale Prävalenz zu berücksichtigen. Testungen sollten niederschwellig für Betroffene erreichbar sein, und die Befundübermittlung sollte standardisiert digital erfolgen. Die für die Durchführung von Testungen notwendigen personellen und logistischen Ressourcen sollten bereitgestellt und Zuständigkeiten durch die Leitenden der Einrichtungen eindeutig geregelt werden. Dabei sollte die Finanzierung der Teststrategie nicht zu Lasten der Unternehmen gehen.

Aktuell werden SARS-CoV-2-Testungen in Deutschland durch die jeweils regional gültige Coronavirus-Testverordnung (TestV) in Verbindung mit der Nationalen Teststrategie [5] geregelt. Die TestV (Stand 21.09.2021) hat dabei maßgeblichen Bezug zum Infektionsschutzgesetz (IfSG) mit dem Ziel, Infektionsketten in der Bevölkerung zu unterbrechen [6]. Für Unternehmen nach §§ 23 und 36 IfSG gilt inzwischen explizit, dass asymptomatische Beschäftigte Anspruch auf Testungen haben, um die Verbreitung des SARS-CoV2-Coronavirus zu verhüten (§ 4 TestV) [6, 7]. Anspruch auf Testung haben Beschäftigte dieser Unternehmen auch nach Auftreten von Infektionen (§ 3) [6]. An der Schnittstelle zwischen IfSG und unternehmerischem Interesse sollen SARS-CoV-2-Infektionen sowohl bei Patient*innen als auch Mitarbeiter*innen verhindert werden.

Bei der Umsetzung von SARS-CoV-2-Testkonzepten in Betrieben kommen allerdings weitere ranggleiche Gesetze und Verordnungen zur Anwendung. Während das IfSG den Bevölkerungsschutz zum Ziel hat, werden über die Arbeitsschutzgesetzgebung weitere Aufgaben für Arbeitgeber AG formuliert, die im Besonderen den Schutz der Beschäftigten sicherstellen sollen.

Die SARS-CoV-2-Arbeitsschutzverordnung (Corona-ArbSchV) vom 21.01.2021 konkretisierte zwar das Arbeitsschutzgesetz (ArbSchG) hinsichtlich der Durchführung von Tests in Bezug auf einen direkten Erregernachweis des Virus in allen Unternehmen [8], stellte dabei aber keinen Bezug zur konkreten tätigkeitsbedingten Infektionsgefährdung her, wie in § 5 ArbSchG eigentlich vorgesehen [8]. Das Ziel war es hier, generell das betriebliche Infektionsrisiko zu mindern (§ 4) [8]. Die dazugehörige Arbeitsschutzregel vom 07.05.2021 wies erstmals auf Überschneidungen hin: „Während der Epidemie überschneiden sich im Betrieb und in den Einrichtungen Anforderungen des bevölkerungsbezogenen Infektionsschutzes mit Maßnahmen des Arbeitsschutzes“ [9]. Eine Konkretisierung der Testangebotspflicht für AG wurde hier allerdings auch nicht vorgenommen.

Aktuell lassen sich demnach keine Testanlässe allein zum Schutz für Beschäftigte ableiten, wiewohl die Grenzen zwischen Arbeitsschutz und Bevölkerungsschutz beim Thema SARS-CoV-2-Testungen verschwimmen. Für Tätigkeiten mit Patient*innenkontakt werden andererseits AG-Pflichten, wie die Veranlassung betriebsärztlicher Vorsorge z. B. nach Biostoffverordnung, sehr klar formuliert. Dies trifft ebenfalls auf das Unfallversicherungsrecht zu, wenn es um die Anerkennung einer Berufskrankheit nach versicherungsrechtlich relevantem Kontakt zum SARS-CoV-2-Erreger geht.

## Zielstellung

Vor dem Hintergrund erlebter Hindernisse bei einer adäquaten Umsetzung gesetzlicher Vorgaben in regelkonforme und rechtssichere Prozesse auf betrieblicher Ebene ist es das Ziel der vorliegenden Untersuchung, insbesondere Vertreter*innen der Gesetzgeberseite mit betrieblichen Akteur*innen und medizinischen Expert*innen zusammenzubringen, um gemeinsam hemmende bzw. fördernde Faktoren für die Steuerung und Optimierung von effektiven und effizienten betrieblichen Testkonzepten herauszuarbeiten und Lösungsstrategien zu entwerfen. Diese erarbeiteten Lösungsstrategien sollen dann zu einem verständlichen Leitfaden im Sinne einer Mustervorlage gebündelt werden.

Die vorliegende Arbeit ist Teil der Able-SOP-Screen-Forschungsarbeit im Rahmen des Verbundprojekts egePan Unimed – Entwicklung, Testung und Implementierung von regional adaptiven Versorgungsstrukturen und Prozessen für ein evidenzgeleitetes Pandemiemanagement, koordiniert durch die Universitätsmedizin Deutschlands (Förderkennzeichen 01KX2021 egePan Unimed). Die vorliegende Studie fokussiert ausschließlich asymptomatische Testungen.

## Methodik

Die Darstellung der verschiedenen Richtlinien sowie der S1-Leitlinie der DIVI zeigen, dass die Beschäftigtentestung einerseits ein Prozess ist, an dem ein breites Spektrum von innerbetrieblichen Agierenden beteiligt sein muss, andererseits verschiedenste Einflussfaktoren – darunter etwa die Definition von Verantwortlichkeiten und die Etablierung von Finanzierungskonzepten – berücksichtigt werden müssen, um Teststrategien erfolgreich implementieren zu können [10]. Diese komplexen Szenarien sind kennzeichnend für Implementierungsprozesse aller Art, weshalb die Implementierungsforschung zum besseren Verständnis dieser Komplexität häufig auf qualitative Studiendesigns setzt [11].

### Studiendesign

Die Autor*innen der vorliegenden Studie haben sich für eine Fokusgruppendiskussion entschieden, um die verschiedenen Teilnehmenden in einen Austausch zu den aus ihren fachlichen oder Interessen geleiteten Meinungen und Sichtweisen zu bringen und so explorativ Informationen auf verschiedenen Ebenen über hemmende und fördernde Faktoren von effektiven und effizienten SARS-CoV-2-Testkonzepten in Einrichtungen des Gesundheitswesens zu gewinnen.

Die Forschungsarbeit übernahm ein multidisziplinäres Team mit arbeitsmedizinischem (SL), medizinischem (RH), gesellschaftswissenschaftlichem (AH), psychologischem (AW) und betriebswirtschaftlichem (LB) akademischem Hintergrund. Auf diese Weise konnte der Einfluss persönlicher Meinungen und fachlicher Prägungen geringgehalten sowie die Validität der Ergebnisse erhöht werden.

### Dokumentenanalyse

**Figure 1:**
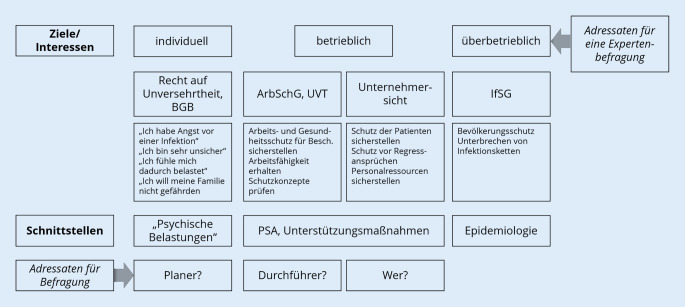

Zur Vorbereitung der Fokusgruppendiskussion wurde in mehreren Sitzungen das Ausgangsmaterial im Sinne einer Informations- oder Faktensammlung in einer Präsentation zusammengestellt. Besprechungsgrundlage bildete die Sichtung aller zu dem Zeitpunkt (Dezember 2020) geltenden Gesetze, Verordnungen und Verfügungen, die für die Ableitung von SARS-CoV-2-Testkonzepten in Betrieben beachtet werden mussten (Abb. 1).

In den Sitzungen wurden schließlich iterativ vier Kategorien identifiziert, in denen verschiedene Handlungsfelder auf überbetrieblicher sowie betrieblicher Ebene aufgrund eigener Erfahrung bereits benennbar waren sowie weitere vermutet wurden. Ihnen konnten Leitfragen zugeordnet werden, die für die Moderation der Fokusgruppendiskussion aufgearbeitet wurden. Diese vier Kategorien bildeten einen analytischen Rahmen, von welchem aus weitere Faktoren zur Erreichung der Zielstellung untersucht werden sollten. Sitzungsinhalte sind stichwortartig dokumentiert, die Ergebnisse der Sitzungen sind in den datierten PowerPoint-Präsentationen nachvollziehbar.

Die Unterscheidung der Zielrichtungen für Testkonzepte ist maßgeblich für nachfolgend im Betrieb etablierte Prozessabläufe einschließlich der Klärung der Frage nach dem zuständigen Kostenträger. Anlässe bzw. Ziele für Beschäftigtentestungen sollten grundsätzlich und eindeutig gesetzlichen Vorgaben folgen (ArbSchG, IfSG) und sich den entsprechenden Verordnungen zuordnen lassen (Tab. 1).

**Table 1:** 

	Individuell	Betrieblich: Beschäftigtenschutz	Betrieblich: Patient*innenschutz, Schutz vor nosokomialen Infektionen	Überbetrieblich: Bevölkerungsschutz, Schutz vor nosokomialen Infektionen
Anlass	Eigeninteresse Unterstützung der Schutzziele des AG Unterstützung der Schutzziele für die Gesellschaft	Verpflichtung der AG zur Umsetzung des Arbeits- und Gesundheitsschutzes für Beschäftigte	Vordergründiges AG-Interesse Übertragungen von Infektionen von Beschäftigten auf Patient*innen sowie innerhalb der Belegschaft verhindern	Unterstützung der Schutzziele für die Gesellschaft Übertragungen von Infektionen auf Patient*innen sowie innerhalb von Personengruppen verhindern
Gesetzliche Grundlagen	Grundgesetz Art. 2 Rechte und Pflichten für Beschäftigte nach ArbSchG § 15 Pflichten als Bürger*in nach IfSG	ArbSchG Regelwerk der Unfallversicherungsträger (UVT)	Unternehmerische Interessen als Schutz vor Regressansprüchen und Personalausfall Im Weiteren nach IfSG und entsprechende Verordnung^a^, welche u. a. auf den Schutz der Patient*innen abstellt Außerhalb der Arbeitsschutzgesetzgebung	IfSG Dazugehörige Verordnungen
Testkonzept	Servicetestung/Angebot durch AG Kein besonderes Risiko durch Tätigkeit Bei Entfallen von Bürger*innentestungen AG-Angebot von Selbsttests Bei positivem Testergebnis kann eine Beratung bei Betriebsärzt*innen erfolgen (→ *Leaving*, Abb. 5)	Risiko durch Tätigkeit Regelmäßige Testung i. S. eines Screenings Kontaktpersonenermittlung und ggf. Umgebungsuntersuchung nach Kontakt zu positiven Patient*innen Einbeziehung der Betriebsärzt*innen Bestandteil der arbeitsmedizinischen Vorsorge PCR oder Schnelltest Regelhafte Beratungsleistung der Betriebsärzt*innen (→ *Institutional*, Abb. 5)	Eingangstest Außerhalb der Arbeitsschutzgesetzgebung erhöhtes Risiko durch Übertragung von Infektionen von Beschäftigten auf Patient*innen; besonders vulnerable Patient*innengruppen Eignungsfragestellung des AG Ergebnisse der Untersuchungen gehen an AG Einbeziehung der Betriebsärzt*innen unter Beachtung vorgenannter Aspekte möglich PCR oder Schnelltest (→ *Coming*, Abb. 5)	Bürger*innentestungen, Selbsttest Kontaktpersonenermittlung und ggf. Umgebungsuntersuchung nach Kontakt zu positiv getesteten Personen im betrieblichen Umfeld PCR oder Schnelltest (→ *Public*, Abb. 5)
Angebot oder Pflicht	Angebot durch AG Freiwillige Inanspruchnahme durch Beschäftigte	Pflichtangebot durch AG Freiwillige Inanspruchnahme durch Beschäftigte	Anordnung des AG verpflichtende Umsetzung durch Beschäftigte	Verpflichtung durch Gesetzgebung Verpflichtende Umsetzung und Duldung durch Beschäftigte
Zuständigkeit für Testungen	Eigenverantwortliche Inanspruchnahme der Testangebote	Als betriebsärztliche Aufgabe denkbar	Durch AG zu definieren, primär keine betriebsärztliche Aufgabe	Gesundheitsamt In der Regel Auftragserteilung an AG
Kostenträger	AG ggf. Bund/Länder ggf. zu Lasten der gesetzlichen Krankenversicherung (GKV)	AG ggf. Unterstützung durch UVT	AG je nach Corona-Virus-TestV kommt eine Beteiligung des Bundes/des Landes in Betracht	Öffentlicher Gesundheitsdienst, Gesundheitsämter Je nach Corona-Virus-TestV kommt eine Beteiligung des Bundes/des Landes in Betracht
Beschäftigtendatenschutz	Ergebnisse aus freiwilligen Tests können mit Betriebsärzt*innen besprochen, dürfen aber nicht für AG, Personalverantwortliche oder weitere Dritte einsehbar sein	Ergebnisse aus Tests im Sinne der arbeitsmedizinischen Vorsorge dürfen mit Betriebsärzt*innen besprochen, aber nicht für AG, Personalverantwortliche oder weitere Dritte einsehbar sein	Ergebnisse aus Tests dienen der Eignungsfeststellung für die Ausführung der Tätigkeit sie müssen dem AG bekannt sein die Freigabe von Beschäftigtendaten muss gesondert vereinbart werden	Ergebnisse aus Bürgertests sowie nach Umgebungsuntersuchungen mit Beschäftigten als Indexfall können mit Betriebsärzt*innen besprochen, dürfen aber nicht für AG, Personalverantwortliche oder weitere Dritte einsehbar sein
Meldepflichten	Meldepflichten nach IfSG			

*AG* Arbeitgeber

^a^Verordnung zum Anspruch auf Testung in Bezug auf einen direkten Erregernachweis des Coronavirus SARS-CoV‑2 (Coronavirus-Testverordnung – TestV); V. v. 14.10.2020 BAnz AT 14.10.2020 V1; aufgehoben durch § 17 V. v. 30. November 2020 BAnz AT 01.12.2020 V1

### Rekrutierung

Ziel war es, eine ausbalancierte, multidisziplinäre Fokusgruppe mit Vertreter*innen möglichst vieler für die Thematik relevanter Entscheider*innen in einen Austausch miteinander zu bringen, um eine holistische Betrachtung der Schnittmengen und Divergenzen anstreben zu können.

Zur geplanten Fokusgruppendiskussion wurden 84 Anschreiben an das BMAS, das BMG, den Ausschuss Arbeitsmedizin der BAuA, DGAUM, VDBW, DGUV, VDSi, VUD, den GKV-Spitzenverband^1^, den Bundesverband Öffentlicher Gesundheitsdienst, die Kommission für Krankenhaushygiene und Infektionsprävention, die Gesellschaft für Virologie, die Gesundheitsministerien der Bundesländer, die Deutsche Krankenhausgesellschaft sowie an die leitenden Betriebsärzt*innen der Universitätskliniken Deutschlands per Mail versendet. Schließlich konnten neun Teilnehmende gewonnen werden. Mit der finalen Zusage wurde die Rekrutierung abgeschlossen.

Die Teilnehmenden wurden über das Teilprojekt sowie dessen Einbindung in die egePan-Verbundforschung informiert und erteilten schriftlich ihre Einwilligung zur Audioaufzeichnung der Diskussion sowie die Erhebung und Verarbeitung erhobener Daten in pseudonymisierter Form.

### Durchführung der Fokusgruppendiskussion

Die Fokusgruppendiskussion erfolgte vor dem Hintergrund pandemiebedingter Beschränkungen sowie aus organisatorischen Gründen über das Online-Videokonferenzsystem Zoom am 16.12.2020. Die Teilnehmenden wurden gebeten, mit dauerhaft eingeschalteter Kamera und Mikrofon an der Diskussion teilzunehmen. Die Moderation der Fokusgruppendiskussion wurde auf der Grundlage der zusammengestellten Leitfragen und unter Beachtung der Anforderungen an eine Moderation von der Studienleiterin selbst übernommen (SL). Zwei wissenschaftliche Mitarbeiterinnen des Projekts erstellten Feldnotizen (RH, AH). Die Teilnehmenden wurden noch einmal zur audiodigitalen Aufzeichnung sowie zum Datenschutz aufgeklärt.

Zu Beginn der Fokusgruppendiskussion wurde den Teilnehmenden mit Hilfe der über den Bildschirm geteilten Darstellung der Nationalen Teststrategie vom 14.10.2020 der Ist-Stand der Strategie zusammengefasst. Anschließend wurde das Forschungsziel erneut dargelegt. Die anschließende Diskussion wurde anhand weniger Leitfragen durch die Moderatorin angestoßen und gesteuert, um auf konkrete Aussagen zu erkannten Hindernissen und förderlichen Faktoren für die Ableitung von Testkonzepten zu fokussieren. Den Teilnehmenden wurde grundsätzlich die Möglichkeit eingeräumt, hierzu auch Verfahren zu beschreiben, die sich aus ihrer Sicht als Best Practices bewährt haben.

Die Fokusgruppendiskussion wurde zwei Stunden lang geführt. In die freie Rede der Antwortenden wurde nicht eingegriffen. Vor dem Wechsel in eine weitere kategorial bezogene Frage bat die Moderatorin regelmäßig um ausstehende Statements.

### Datenanalyse

Der Durchführung der Fokusgruppe folgte eine Transkription sowohl der Leitfragen als auch aller Diskussionsinhalte mit dem Gesprächsanalytischen Transkriptionssystem GAT 2 unter Nutzung des Transkriptionsprogramms f4transkript (dr. dresing & pehl GmbH, Marburg, Deutschland), die anschließende Analyse erfolgte mit Hilfe der Software MAXQDA 2018 (VERBI Software GmbH, Berlin, Deutschland). Das Ausgangsmaterial lag schließlich in Textform vor.

Für die Auswertung des Transkripts wurde ein komplementäres Vorgehen gewählt. So erfolgte die Analyse durch eine Mitarbeiterin (AH) nach der deduktiven Kategorienanwendung, um zu überprüfen, welche Gesprächsinhalte nicht den vorab gebildeten Kategorien zuzuordnen waren. Zwei andere Mitarbeitende (AW und LB) analysierten das Ausgangsmaterial nach der induktiven Kategorienbildung, um zu überprüfen, welche Handlungsfelder und konkreten Handlungsanweisungen sich hier ergeben und welche Faktoren sich als hemmend und fördernd herausgestellt haben. Beide Analyseverfahren folgten den Regeln der qualitativen Datenanalyse nach Mayring [12]. Die Ergebnisse wurden jeweils in einem Kodierungssystem dokumentiert und erst nach der Fertigstellung miteinander verglichen und zusammengeführt. Bei Abweichungen, meist aufgrund inhaltlicher Überschneidung mancher Kategorien, erfolgte eine Konsensbildung im Austausch aller an der Analyse beteiligten Mitarbeiter*innen (AW, LB, AH) sowie der Studienleiterin (SL).

Alle Ergebnisse sind mit Ankerbeispielen belastbar.

## Ergebnisse

Um die Anonymität der Fokusgruppenteilnehmenden sicherzustellen, wurden für die nachfolgende Ergebnisdarstellung die Teilnehmenden vier Gruppen zugeordnet:

1. zwei Praktiker*innen auf betrieblicher Ebene (Fachärzt*innen für Arbeitsmedizin, Betriebsärzt*innen) – BÄ
2. ein(e) Arbeitgebervertreter*in (Personalverantwortliche, Unternehmensleiter*innen) – AG
3. drei Vertreter*innen der medizinischen Fachwissenschaft (Arbeitsmedizin, Virologie, Krankenhaushygiene, Infektiologie) – FA
4. zwei Vertreter*innen politischer Gremien, Behörden und Dachverbänden (Gesundheitsministerien, Gesetzliche Unfallversicherer, Krankenversicherer, Krankenhausgesellschaft) – G

### Analyse der Fokusgruppendiskussion

**Figure 2:**
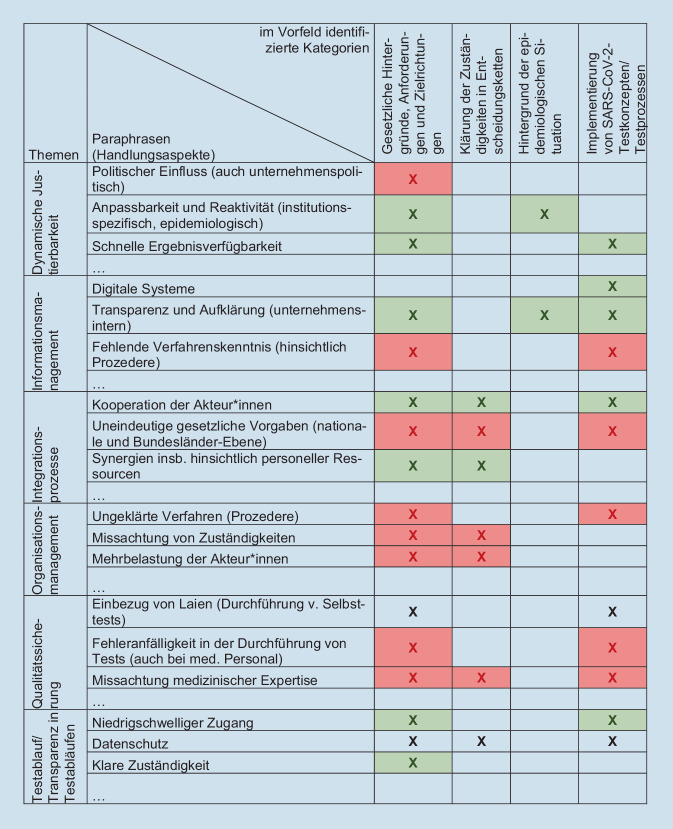

Durch induktive Kategorienbildung konnten 52 Paraphrasen identifiziert werden, die sechs verschiedenen Überkategorien zugeordnet wurden (Abb. 2). Die Überprüfung nach der deduktiven Kategorienanwendung ergab keine neuen Handlungsebenen zu den im Vorfeld festgelegten Kategorien.

### Meistdiskutierte Themenbereiche

**Figure 3:**
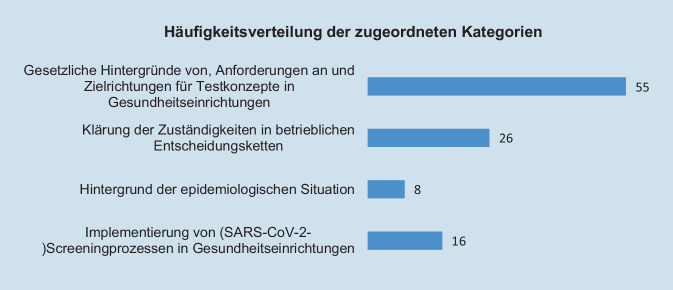

Die häufigsten Wortmeldungen konnten der Kategorie *Gesetzliche Hintergründe von, Anforderungen an und Zielrichtungen für Testkonzepte in Gesundheitseinrichtungen* zugeordnet werden (52,4 % der Antworten). Weitere 24,8 % der kodierten Antworten ließen sich den Fragen zu *Zuständigkeiten für Umsetzung in betrieblichen Entscheidungsketten* zuordnen. 15,2 % der analysierten Gesprächsinhalte bezogen sich auf Fragen zur *Implementierung von SARS-CoV-2-Testkonzepten/Testprozessen*. Bei 7,7 % wurde die Berücksichtigung der epidemiologischen Situation diskutiert (Abb. 3).

**Figure 4:**
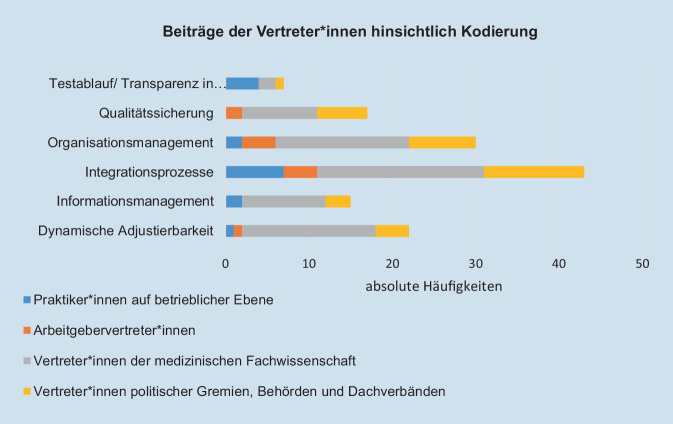

Die Häufigkeit der Wortmeldungen je Expert*innenhintergrund und zuordenbare Kategorie bzw. Leitfrage war zugunsten praxisnaher Expert*innen (Praktiker*innen auf betrieblicher Ebene, AG-Vertreter*innen und Vertreter*innen der medizinischen Fachwissenschaft) verteilt (Abb. 4).

#### Gesetzliche Hintergründe von, Anforderungen an und Zielrichtungen von SARS-CoV-2-Testkonzepten in Gesundheitseinrichtungen

Dieser Kategorie wurden die meisten der paraphrasierten Textinhalte zugeordnet. Paraphrasen wurden am häufigsten zu „Integrationsprozesse“ (11 Paraphrasen) und „Dynamische Justierbarkeit“ (7 Paraphrasen) kodiert.

In der Diskussion bestand weitgehend Einigkeit darüber, dass Testangebote verschiedenen Zielrichtungen und unterschiedlichen gesetzlichen Grundlagen folgen. Es müsse unterschieden werden, ob das Testkonzept beispielsweise den Beschäftigtenschutz im Fokus hat und entsprechende Prozesse das ArbSchG berücksichtigen müssen oder ob im Sinne des Patient*innen- bzw. Drittschutzes das IfSG Anwendung findet. Eine konsequente und regelkonforme Anwendung der Arbeitsschutzgesetzgebung durch die AG stehe im Konflikt mit einigen Inhalten aus der Infektionsschutzgesetzgebung und wirke sich limitierend auf die reibungsfreie Umsetzung von Testkonzepten auf betrieblicher Ebene aus.

„Ich glaube einen Punkt, den wir vorschalten müssen [ist], in welcher Welt wir uns bewegen, wenn ich das mal so ausdrücken darf. Sind wir im Bereich des Infektionsschutzgesetzes, das ist allgemeiner Bevölkerungsschutz, oder sind wir im Bereich des Arbeitsschutzes, der möglicherweisen ganz andere Kriterien verlangt als Infektionsschutz oder Infektionsschutzgesetz.“ (FA, Pos. 31)

Die nachfolgenden Zitate aus der Fokusgruppendiskussion wurden exemplarisch für einige andere ähnliche Aussagen zu notwendigen „Integrationsprozessen“ herangezogen.

Diskussionsteilnehmende äußerten die Erwartung, dass in Anbetracht der sehr komplexen Zusammenhänge zunächst Abstimmungen auf Gesetzgeberebene erfolgen müssen, um die nachfolgende Umsetzung der Gesetze und Verordnungen sicherzustellen.

„Das ist eine politische Diskussion, die muss eigentlich zwischen Bundesgesundheitsministerium, das für das Infektionsschutzgesetz zuständig ist und dem Bundesarbeitsministerium für den Arbeitsschutz auf Bundesebene letztendlich insbesondere bei dem föderalen Staat auf Landesebene geführt werden.“ (FA, Pos. 52)

Auf Landesebene sei es dann z. B. konkret vorstellbar, dass mit Vertreter*innen aus Politik, Interessen- und Fachvertretungen Fragen geklärt und Lösungen vorbereitet werden können. Hier seien auch unbedingt Vertreter der gesetzlichen UVT mit einzubeziehen.

„Also wenn ich ein großes Klinikum habe, wie zum Beispiel jetzt Universitätsmedizin Dresden, dann würde ich doch von Seiten des Arbeitgebers zusammen mit der Arbeitnehmervertretung an die beiden Ministerien und die BGW als Berufsgenossenschaft herantreten und sagen: Wir machen mal einen runden Tisch, da setzen wir uns zusammen und schauen, wie man dieses Thema hier lösen kann.“ (FA, Pos. 62)

In der Diskussion wurde zudem auf die Volatilität rechtlicher Vorgaben und Handlungsrahmen eingegangen und die damit verbundenen besonderen Anforderungen an die Flexibilität bestehender Strukturen und Prozesse für betriebliche SARS-CoV-2-Testkonzepte herausgestellt. Auf diese *dynamische Justierbarkeit* der Systeme wurde in der Diskussionsrunde mehrfach hingewiesen.

„Das heißt, es gibt dann irgendwelche Verordnungen im Land, wo es heißt, ab der Infektionszahl, ich sag jetzt mal 50 oder 25 pro 1000 und so weiter, passiert irgendwas. Und dann entgleitet der Politik die Infektionszahl, dann wird das alles in die Tonne geklopft und es werden neue Zahlen auf den Markt geworfen. Und das ist für die Bevölkerung, auch für die Fachleute, absolut nicht nachvollziehbar.“ (FA, Pos. 151)

Des Weiteren würden Betriebsstrukturen die Ableitung von Konzepten und Prozessen mitbestimmen. Nicht jede Einrichtung zur Patient*innenversorgung verfüge über dieselben Strukturen wie beispielsweise ein Universitätsklinikum.

„Wir waren glaube ich vorhin schon mal an so einem Punkt, dass wir generelle Konzepte relativ schwierig sehen, sondern das muss der Institution angepasst werden.“ (FA, Pos. 107)

#### Klärung von Zuständigkeiten auf betrieblicher Ebene

Paraphrasierte Aussagen ließen sich hier am häufigsten zu „Integrationsprozesse“ sowie „Organisationsmanagement“ kodieren.

„[…] ich glaube, dass es total wichtig ist, unabhängig von den Kernzuständigkeiten, die ja gleichbleiben, dass es zusammenfließt, weil das Thema Drittschutz, Mitarbeiterschutz, wenn ich mir ein Krankenhaus betrachte, wo ich mir vorstelle, Mitarbeiter, wenn der jetzt positiv ist, dann steckt er andere Mitarbeiter an.“ (AG, Pos. 40)

Bei der Klärung von Zuständigkeiten auf betrieblicher Ebene sei ebenso die Herausforderung zu bewältigen, dass sich der Schutz der Beschäftigten vor einer Ansteckung durch ihre Tätigkeit und der Schutz Dritter (Patient*innen, Kolleg*innen) nicht immer unabhängig voneinander betrachten ließen. Nachfolgende Beispiele wurden für angeführte „Integrationsprozesse“ innerhalb betrieblicher Strukturen ausgewählt.

Es seien Konzepte wünschenswert, die weder die Betroffenen noch die Ausführenden oder die Betriebsabläufe belasten. Dafür müssten alle Beteiligten für eine Zielerreichung zusammenarbeiten.

„[…] der Betrieb muss ja das Testkonzept erstellen, dafür hat er ja dann auch die Freiheiten und den Handlungsspielraum das eben all diese verschiedenen Bereiche dann miteinander Hand in Hand gehen müssen und das auch abdecken müssen. Also was den Infektionsschutz betrifft, was den Arbeitsschutz betrifft und was natürlich jetzt dann halt auch den Schutz der Bewohnerinnen und Bewohner dann halt auch betrifft, oder der Besucher und Besucherinnen.“ (G, Pos. 83)

Es sei außerdem wichtig, alle weiteren notwendigen Fachbereiche im Betrieb einzubeziehen und deren Aufgaben festzulegen. Außer den medizinischen Fachbereichen seien insbesondere bspw. auch IT-Fachleute zu beauftragen, die Dateninfrastruktur sicherzustellen.

„Also ich denke, so Testkonzepte sind ein Zusammenspiel von mehreren Akteuren, die [sich] zusammensetzen müssen und einen vernünftigen Plan [entwickeln], […] da muss einmal der Labordienstleister drin sein, […]. Dann ist sicherlich die Betriebsmedizin wichtig, […], dieses ganze Thema Daten, also Datenübertragung, EDV-Befund, Rückübermittlung, also […], die IT muss irgendwo auch mit dabei sein.“ (FA, Pos. 127)

Eine Klärung und Festlegung der Zuständigkeiten ließe neben der Kalkulation des Arbeitsaufwandes und der zu planenden Ressourcen in den einzelnen Fachbereichen auch die Verteilung der dazu notwendigen Finanzmittel zu.

„Dann ist die Frage, aus welcher Kasse wird das Ganze bezahlt. Das heißt, sie haben sicherlich ja auch in Ihrer Klinik für die Betriebsärzte ein Budget und ein Budget für die Hygiene, die hart umkämpft sind und da kann nicht der eine für den anderen – kann er natürlich schon aber da gibt’s großen Streit, wenn der eine die Arbeit für den Anderen machen soll.“ (FA, Pos. 41)

Nachfolgende Zitate wurden unter „Organisationsmanagement“ kodiert. Sie wurden als Beispiele dafür ausgewählt, dass trotz interdisziplinärer Absprachen auf betrieblicher Ebene falsche Schlüsse für Zuständigkeiten gezogen wurden.

Bei der Entscheidung, wer die SARS-CoV-2-PCR-Testungen bei Beschäftigten vornehmen sollte, bestünden trotzdem weiterhin Unklarheiten. So würden unabhängig von der Zielrichtung der Testkonzepte immer wieder Betriebsärzt*innen genannt. Dabei werde völlig ausgeblendet, dass deren Personalstrukturen klaren Aufgabenstellungen und Personalressourcenzuweisungen folgen.

Es bestünde zum Beispiel die falsche Annahme, dass Abstrichuntersuchungen bei Beschäftigten auch dann eine regelhafte Aufgabe für Betriebsärzt*innen sei, wenn der auslösende Anlass nicht der Tätigkeitsbezug ist. Die durch sie auf mehreren Ebenen erlebten hemmenden Umstände für eine zielführende Umsetzung von Teststrategien konnte von Seiten der Gesetzgebervertretung scheinbar nicht nachvollzogen werden.

„Es ist so, dass zumindest von manchen Personalabteilungen sicherlich immer, wenn das Wort Mitarbeiter fällt, gedacht wird, dafür ist der Betriebsarzt zuständig, auch wenn, sagen wir mal manchmal die Mitarbeiterscreenings vielleicht darauf hin ausgerichtet sind, dass es zu einem Drittschutz führt. Also, dass der Patient geschützt ist und hier wäre eigentlich das ein krankenhaushygienisches Problem. Aber wie gesagt, da wird der Betriebsarzt, der praktisch tätige Betriebsarzt eben im Sinne des Infektionsschutzgesetzes genutzt.“ (BÄ, Pos. 39)

Die Übertragung solcher Aufgaben sei nicht nur im falschen Zuständigkeitsbereich, sondern würde aufgrund des immensen Ressourcenaufwands auch dazu beitragen, dass Betriebsärzt*innen nicht ihren eigentlichen gesetzlichen Aufgabenstellungen folgen können.

„Natürlich kann ich den [Betriebsarzt] nutzen, um Abstriche zu machen und Schnelltests und Ähnliches, aber dann kommt der seiner eigentlichen Aufgabe nicht mehr nach. Und wenn wir dann einen schweren Unfall haben und sowohl wir als DGUV als auch der staatliche Arbeitsschutz vor Ort sind und dann nachfragen, wie konnte das kommen, können Sie sich nicht damit rausreden: Ach wir mussten Infektionsschutz gerecht werden und eventuell Tests abnehmen. Also ich sehe das auch ein bisschen kritisch. Sicherlich kann ich es vertreten, ein paar Abstriche mehr zu machen, aber die Betriebsärzte originär dafür einzusetzen, hätten wir auch schon was dagegen“. (G, Pos. 43)

#### Implementierung von SARS-CoV-2-Testkonzepten/Testprozessen

Paraphrasierte Aussagen ließen sich hier am häufigsten zu „Qualitätssicherung“ sowie „Integrationsprozesse“ kodieren. Weitere wichtige Aspekte ließen sich den Kodes „Transparenz in Testprozessen“ sowie „Informationsmanagement“ zuordnen.

Es sei sehr kritisch zu betrachten, dass im Rahmen der Einführung von Antigen-Schnelltests zunehmend Abstriche durch nichtärztliches Personal durchgeführt würden bzw. durch Personal, das nicht hinreichend in die konkrete Technik eingewiesen sei. Das berichteten sogar Vertreter*innen aus den Gesundheitsberufen aus eigener Erfahrung. Dieser Punkt wiege umso schwerer vor dem Hintergrund der Ausweitung der Testangebote in allen Betrieben bzw. der geplanten Einführung von Selbsttests.

„[…] da hat man also auch gemerkt, dass selbst geschulte Mitarbeiter in den Ambulanzen also sehr unterschiedlich die Abstriche entnehmen und eben zum Teil auch wirklich falsch.“ (FA, Pos. 146)

Folgende Aussagen konnten dem Kode „Qualitätssicherung“ in der Kategorie *Implementierung von SARS-Cov-2-Testkonzepten/Testprozessen *zugeordnet werden.

Ebenso würden sich IT-Schnittstellenprobleme in komplexen Prozessabläufen auf die Qualität ebensolcher auswirken. Befunderfassung und -management bestimmten maßgeblich den Erfolg von Testkonzepten. Zahlreiche, parallellaufende Prozesse bei der Bewältigung der Anforderungen, wie zum Beispiel Beschäftigtentestungen und Kontaktpersonennachverfolgungen mit einer entsprechend enormen Menge an administrativen Aufgaben und anfallenden Befunden seien ohne den Einsatz von aufeinander abgestimmten digitalen Systemen nicht effizient und womöglich nicht effektiv umsetzbar. Eine funktionierende digitale Vernetzung der beteiligten medizinischen Fachabteilungen habe durch den Informationsaustausch erhebliches Potenzial in der Pandemiebekämpfung.

„Es gibt unterschiedliche Systeme, wir haben das Betriebsarztsystem, wir haben die Krankenakte, wir haben das Laborsystem. Manches arbeitet miteinander, manches arbeitet nicht miteinander. Und wenn man auf der Ebene eben wesentlich bessere Verknüpfungen und Tools schafft, also wirklich massiv in digitale Lösungen investiert, dann kann man glaube [ich] viele dieser Fragen ganz befriedigend, zufriedenstellend lösen.“ (FA, Pos. 159)

Für erfolgreiche Implementierungsstrategien seien fortwährende Abstimmungsprozesse zwischen den beteiligten Fachbereichen unbedingt erforderlich. Diese Beispiele wurden den dafür notwendigen „Integrationsprozessen“ zugeordnet.

„Ich glaub[e], dass es ganz viel darauf ankommt, dass das Zusammenspiel passt: betriebsärztlicher Dienst und Krankenhaushygiene, Virologie aber auch dem Personalbereich.“ (AG, Pos. 10)

Große Unsicherheit oder sogar massive Fehlannahmen beträfen die Freiwilligkeit der Inanspruchnahme von Testangeboten. So seien manche AG der Meinung, sie können ohne eine eindeutige Regelung Testpflichten für Beschäftigte einführen. Es sei nicht allen klar, dass eine solche Verpflichtung nur auf der Grundlage von eindeutigen gesetzlichen Regelungen möglich wäre.

„Ein weiterer Punkt ist auch die Freiwilligkeit der Beschäftigten. Was wollen die? Wir können Sie definitiv nicht zwingen, jetzt an irgendwelchen Abstrichen teilzunehmen und dann welche Informationen bekommt der Arbeitgeber.“ (G, Pos. 45)

Um den Zugang zu Beratungs- und Testangeboten im Zusammenhang mit verschiedenen Anlässen für Beschäftigte tatsächlich sicherzustellen, seien entsprechende Versorgungsmodelle durchaus vorstellbar.

„Wir hatten uns ja ganz praktische Umsetzungen überlegt, dass es in Absprache mit dem betriebsärztlichen Dienst gegebenenfalls eine Art Corona-Zentrum gibt, in dem quasi Zusatzaufgaben, die jetzt auf verschiedene Leute zukommen, also Screenings, Beratung, Umgebungsuntersuchungen, dass die dort abgearbeitet werden.“ (BÄ, Pos. 71)

Des Weiteren müssten Testangebote so platziert werden, dass Beschäftigte sie möglichst niedrigschwellig und kurzfristig in Anspruch nehmen könnten. Sie müssten wissen, wohin sie sich wann wenden können und wie alle weiteren Prozesse geregelt sind. Nachfolgendes Beispiel wurde dem Code „Transparenz in Testprozessen“ zugeordnet.

„[…] die Hürden für so eine Testung müssten, wenn es für die Mitarbeiter wäre, […] sehr niedrig sein […].“ (BÄ, Pos. 77)

Die Bereitstellung von Informationen sowie die Möglichkeit, sich individuell beraten zu lassen, seien wesentliche Bestandteile eines niederschwelligen Angebots an Beschäftigte. Im Rahmen der Implementierungsstrategien wurde das nachfolgende Beispiel dem Code „Informationsmanagement“ zugeordnet.

„Es entstehen unendlich viele Fragen, an die man vorher gar nicht gedacht hat, die die Mitarbeiter haben, die zu Verunsicherung führen, zu Unruhe führen, zu vielleicht auch unvorsichtigem Verhalten, sodass also sowas ganz, ganz wichtig ist im Alltag, dass die Leute wirklich gut informiert werden und auch Möglichkeit haben, sich niederschwellig zu informieren in der Einrichtung.“ (BÄ, Pos. 85)

#### Epidemiologische Situation („Expert*innenmeinung“)

Paraphrasierte Aussagen ließen sich hier am häufigsten zu „Informationsmanagement“ sowie „Dynamische Justierbarkeit“ kodieren.

SARS-CoV-2-Testungen seien unbedingt in ein Gesamtkonzept für Infektionsschutzmaßnahmen zu integrieren. So können alle Testkonzepte Infektionen nicht verhindern, sondern lediglich einen Beitrag zur Aufdeckung von Infektionsfällen leisten. Für eine Verhinderung von Infektionsfällen und der Ausbreitung von Infektionen und Erkrankungen müssen weitreichende Schutzmaßnahmen eingeführt und konsequent umgesetzt werden.

Neben der Möglichkeit, eine SARS-CoV-2-Infektion im privaten Umfeld zu erwerben, fände zudem eine Übertragung unter den Beschäftigten in den Pausen- und Sozialräumen statt. Hier sei nach Arbeitsschutzgesetzgebung grundsätzlich kein Auftrag mehr für den Arbeitsschutz abzuleiten – diese Problematik mit entsprechenden Konsequenzen würde den Aufgaben nach der Infektionsschutzgesetzgebung zugeordnet und sei eher organisationaler Natur. Das angeführte Zitat wurde beispielhaft für ein entsprechendes „Informationsmanagement“ herangezogen.

„Das große Problem ist ja eigentlich, dass wir an dem Bereich, wo Arbeitsschutz funktioniert, relativ wenige Infektionen haben. Wo passiert es? Wir hatten in der Klinik in den Sozialräumen das Problem. Die haben sich […] in der Klinik an alles gehalten und dann sind sie in die Pause gegangen, Maske runter, nebeneinandergesetzt, übertreib jetzt und Glühwein getrunken. Und da haben sie sich angesteckt. Also das muss glaub ich auch im Bereich der Informationspolitik in die Köpfe rein. Da muss auch organisatorisch was passieren.“ (FA, Pos. 161)

Mit der Erfahrung aus der zweiten Infektionswelle müsse hinsichtlich der Einfluss- und Beratungsmöglichkeit durch Fachexpert*innen kritisch angemerkt werden, dass diese mit dem Ausmaß des Infektionsgeschehens und einem zunehmenden politischen Druck deutlich sinke. Man nehme die Gefahr hinsichtlich neuer Anordnungen und Maßnahmen wahr, dass diese schwerer vermittelbar würden und die Einrichtungen vor kaum zu bewältigende Aufgaben stellten. Nachfolgendes Zitat wurde aus diesen Gründen als Beispiel für „Dynamische Justierbarkeit“ im Zusammenhang mit der *Epidemiologischen Situation* gewählt.

„[…] man kann zu Beginn der Sache, […] wenn ein gewisser Druck da ist, aber irgendwie ist es einigermaßen beherrschbar, wie das [bei] uns im Frühjahr […] war, da kann man Pläne machen und dann wird auch auf uns, die ich uns mal jetzt Experten nenne, gehört. Wir können mit dezidierten Meinungen vortreten. Da hört man uns zu. Das finden wir klinisch, epidemiologisch, arbeitsmedizinisch wie auch immer sinnvoll. Und wenn aber der Druck steigt und dann auch äußere, sozusagen Druckgeber auf die Leitung dieser Krankenhäuser eben wesentlich stärker werden, dann wird die Expertenmeinung eigentlich immer geringer und es beginnt Aktionismus […].“ (FA, Pos. 95)

Für die Erarbeitung von Testkonzepten sei die Grundkenntnis weiterer biostatistischer Einflussfaktoren zu den Testmethoden selber, aber auch zur Prävalenz der Erkrankung unerlässlich. Die Frage also, was ein Test selber leisten kann, sollte in die Überlegung aufgenommen werden.

„[…] das ist absolut wichtig, dass man tatsächlich Testkonzepte an Testleistungsfähigkeiten anpasst, also, dass nur so ein bisschen Biostatistik eines Tests ist es ja Sensitivität, Spezifizität, Prävalenz der Erkrankung und daran ein Testkonzept ausrichtet. Also was ist der positiv-prädiktive Wert eines Ergebnisses, was ist der negativ-prädiktive Wert eines Tests? […] wenn man den Test sachgerecht einsetzt, muss man sich schon an seiner Leistungsfähigkeit orientieren und auf der Basis in der Gesamtschau ein Testkonzept entwickeln, was wirklich dann stimmig ist, also das sehe ich absolut so, und das ist sicherlich auch geboten, um den Test vernünftig einzusetzen und die Aussagen vor allen Dingen verwertbar zu machen.“ (FA, Pos. 149)

## Diskussion und Implikationen

Die wesentlichen Ergebnisse der vorliegenden Arbeit sind die hohe Übereinstimmung insbesondere der betrieblichen Akteur*innen (Praktiker*innen, AG-Vertreter*innen, Vertreter*innen der medizinischen Fachwissenschaften) bei der Feststellung, dass mit dem IfSG und der Arbeitsschutzgesetzgebung unterschiedliche Zielrichtungen für Testkonzepte verfolgt werden, diese aber für eine Ableitung und Umsetzung von betrieblichen Testkonzepten berücksichtigt werden müssen. Es wird daher von ihnen erwartet, dass bereits in Legislative und Exekutive auf Bundes- und Landesebene Abstimmungen unter Einbeziehung von Interessen- und Fachvertretungen erfolgen. Auf einer betrieblichen Ebene bestimmen die Einbeziehung der für Beschäftigtengesundheit und -datenschutz zuständigen Fachschaften sowie der IT-Fachleute den Erfolg von Testkonzepten maßgeblich.

In der Fokusgruppendiskussion wurde häufig erwähnt, dass betriebliche Akteur*innen Probleme mit der Übersetzung von gesetzlichen Vorgaben in die Praxis erkennen. Hinweise zu dieser Beobachtung gab es bereits vor Corona. In dem Gutachten „Erst Inhalt, dann die Paragrafen“ im Auftrag des Normenkontrollrates der Bundesregierung wird bereits 2019 darauf hingewiesen, dass neben der formalen Güte von Gesetzen diese vor allem praxistauglich sein müssen: „Ein gutes Gesetz […] ist zudem adressatenfreundlich – es verursacht wenig Umsetzungs- und Folgeaufwand für Bürger*innen und Unternehmen. Und es ist vollzugstauglich, so dass die Verwaltung es rechtssicher und kosteneffizient umsetzen kann“ [13]. Mit Bezug zum Thema der vorliegenden Arbeit werden diese diskutierten Inhalte daher einer *überbetrieblichen Entscheidungsebene* zugeordnet.

### Überbetriebliche Entscheidungsebenen

Ein zentrales Ergebnis war die Wahrnehmung insbesondere durch betriebliche Akteur*innen, dass eine gesetzeskonforme Umsetzung der Arbeitsschutzgesetzgebung im Konflikt mit (neuen) Inhalten des IfSG steht. Mangelnde Abstimmung auf bundes- oder länderpolitischer Ebene führt zu Umsetzungsproblemen – nicht nur in Fragen des Beschäftigtendatenschutzes, sondern auch für die Klärung von Zuständigkeiten und entsprechenden Mittelzuweisungen für eine Umsetzung der geforderten Testkonzepte.

Beraterstäbe können Abstimmungsprozesse unterstützen [14]. So sollten Fachexpert*innen der Infektionsschutz- z. B. mit denen der Arbeitsschutzgesetzgebung unbedingt im Austausch stehen, um nachfolgende Konflikte bei der settingbezogenen Umsetzung von Regeln und Verordnungen der jeweiligen Gesetzgebung in Betrieben zu vermeiden. Es sollten weiter Vertreter*innen mit nachweislichem Bezug zur betrieblichen Praxis aufgenommen werden, die in Berufsverbänden und Interessenvertretungen zu finden sind. Zu einem ähnlichen Ergebnis kommen Sell et al. [15], die im Zeitraum bis Juli 2020 die Einberufung von Expert*innen zur Beratung der Bundes- und Landespolitik untersucht haben. Im Ergebnis zeigten sie eine eingeschränkte Interdisziplinarität bei der Zusammensetzung von Expert*innenräten mit einer deutlichen Dominanz biomedizinischer Disziplinen. Die Autor*innen kommen unter anderem zu dem Schluss, dass aufgrund der Komplexität der Auswirkungen von Pandemien auch die Pluralität der wissenschaftlichen Perspektiven sowie die Perspektiven aus der Bevölkerung in die Politikberatung einbezogen werden müssen.

### Entscheidungsprozesse auf betrieblicher Ebene

Die vorliegende Auswertung der Diskussionsinhalte weist auf bestehende Entscheidungs- und Regelungshürden in Bezug auf SARS-CoV-2-Testprozesse/Testkonzepte und deren Implementierung auf betrieblicher Ebene hin. Insbesondere die betrieblichen Akteur*innen beschrieben neben einer empfundenen gleichmachenden und willkürlichen Anwendung der bestehenden Gesetze eine ebensolche Zuweisung oder Anordnung von Aufgaben und Aufträgen auch in den Betrieben selber. Als Gründe hierfür wurden eine generelle Unsicherheit bei der Anwendung von Gesetzen bei gleichzeitig bestehender Herausforderung genannt, die Ziele von Testkonzepten eindeutig einer gesetzlichen Grundlage zuzuordnen. Daneben bestand weitgehende Einigkeit darüber, dass volatile gesetzliche Vorgaben die Anpassung betrieblicher Prozesse in den vorgegebenen zeitlichen Zeitfenstern erschwerten.

Die bereits von der Studiengruppe um Dahmen [14] beschriebenen Herausforderungen hinsichtlich der Klärung von Zuständigkeiten, Strukturen und Prozessen am Beispiel der Organisation konnten auch in dieser Diskussionsrunde bestätigt werden. Wiederholt wechselnde Falldefinitionen, schneller Wandel hinsichtlich empfohlener Schutzmaßnahmen, große Dezentralität und Heterogenität im Öffentlichen Gesundheitsdienst bei der Umsetzung des IfSG sind beispielhaft genannte Herausforderungen, die sich auch auf das Thema Testkonzepte in Betriebe übertragen ließen. Nach Dahmen et al. [14] zeigten sich Herausforderungen im Zusammenhang mit COVID-19 in den genannten Themen überall dort, wo bereits im bisherigen Alltag Verbesserungspotenzial bestand.

Für die Umsetzung von Aufgaben aus Arbeitsschutz‑, Arbeitssicherheits- und IfSG bzw. entsprechend er konkretisierender Verordnungen muss daher auch auf betrieblicher Ebene konsequent Übersetzungsarbeit geleistet werden. Für diese Anforderungen ist die Expertise entsprechender Vertreter*innen im Betrieb unter Einbeziehung interner und/oder externer Sozialpartner*innen (Betriebs- oder Personalrat, UVT, Rentenversicherungsträger) unbedingt notwendig. Zu empfehlen ist die Bildung fester betrieblicher Beratungs- oder Krisenstäbe, in denen diese Expertise gebündelt werden kann. Fachschaften, die als Unterstützer verstanden werden können (Datenschutz, Rechtsabteilung, Kommunikation), sollten außerdem mit einbezogen werden. In Einrichtungen, die nicht jede Expert*innenposition besetzt haben, könnten stellvertretende Fachschaften bestimmte Fragestellungen übernehmen. Einrichtungsübergreifende Netzwerke könnten ebenso bei Entscheidungen und Vorgehen unterstützen. Neben der Klärung der Zuständigkeiten für die Umsetzung und Prozessgestaltung von Testkonzepten muss auch die Frage geklärt werden, wer für welche Kosten aufkommen soll oder muss.

### Klärung von Zuständigkeiten, Prozessgestaltung, Implementierung

Wenn Testanlässe und deren gesetzliche Grundlagen identifiziert sind, können Zuständigkeiten für Organisation und Durchführung von Beschäftigtentestungen formal leichter zugeordnet werden. Bei sich überschneidenden Testzielen können übergreifende Aufgabenstellungen nicht ausgeschlossen werden, was den Vorschlag begründet, eher von Kernzuständigkeiten für Akteur*innen in Prozessen zu sprechen.

Unabhängig davon, welcher Fachgruppe welche konkreten Aufgaben danach für eine gemeinsame Zielerreichung zugeordnet werden, müssen entsprechende Ressourcen zur Verfügung gestellt werden. Weder für Betriebsärzt*innen noch für andere Fachgruppen sind zusätzliche Aufgaben über Wochen oder gar Monate leistbar, ohne von anderen Aufgaben entbunden oder durch zusätzliche Personalzuführung entlastet zu werden.

Insbesondere die betrieblichen Akteur*innen der Fokusgruppe stellten bei dieser Art integrativen betrieblichen Prozessen außerdem heraus, dass IT-gestützte Schnittstellenfunktionen bisher oftmals nicht vorhanden waren. Hier ergeben sich aber Notwendigkeiten, zwischen „Befundentnahmestelle“, Labor, Beschäftigtem und Beratenden ebenso eine funktionierende und den Beschäftigtendatenschutz berücksichtigende Datenstruktur herzustellen sowie gesetzlich geforderte Meldewege zu berücksichtigen.

Die Unterstützung durch IT-Steuerungs- und Controlling-Instrumente ist unentbehrlich. Nicht ohne Grund bezeichnen die Autoren um Caliebe die Corona-Krise in ihrem Beitrag im *Deutschen Ärzteblatt* vom März 2021 als „Missing-Data-Krise“ und beklagen damit die mangelnde Infrastruktur, um „[…] wichtige Daten zum Test- und Infektionsgeschehen und zur Wirksamkeit von Eindämmungs- und Therapiemaßnahmen zu akquirieren und zeitnah verfügbar zu machen“. Die Autor*innen weisen explizit auf die Notwendigkeit hin, dass ohne die Beachtung bestimmter weiterer Einflussfaktoren wie u. a. geografische Herkunft, Testanlass, lokale Prävalenz, Testintensität usw. zielgerichtete, effektive Schutzmaßnahmen nicht abgeleitet werden können [16]. Bereits in einer frühen Phase der COVID-19-Pandemie kommen Trimborn et al. [17] zu dem Schluss, dass Geovisualisierung und Datenintegration gezielte infektionspräventive Maßnahmen wie z. B. Impf- und Aufklärungskampagnen möglich machen. Mehrere Autor*innen stellen die Wichtigkeit eindringlich heraus, neben positiven Testergebnissen auch die Gesamtzahl der durchgeführten Tests zu erfassen. Damit werden genauere Einschätzungen zum tatsächlichen Anstieg an Infektionen möglich sowie ein umfassenderes Bild von der Umsetzung der Teststrategie gewonnen [10, 16–18].

Auf der betrieblichen Ebene bedeutet eine solche Zusammenführung verschiedener Informationen das Vernetzen unterschiedlicher IT-Erfassungssysteme wie bspw. Personal‑, Labor- und arbeitsmedizinische Datenbanken mit individuellen, behördlichen und statistischen Meldesystemen unter Einhaltung des Beschäftigtendatenschutzes. Eine reibungslose Interaktion aller erforderlichen und eingebundenen IT-Systeme ist die Voraussetzung für eine zeitnahe Akquirierung und Analyse von vorhandenen Daten und trägt zudem wesentlich zur Steigerung der Effizienz und Effektivität bei.

### Epidemiologische Situation, biostatistische Einflussgrößen, Arbeitsschutzmaßnahmen

Eine wichtige Erkenntnis der Fokusgruppendiskussion war die übereinstimmende Feststellung, dass betriebliche Testkonzepte lediglich eine ergänzende Maßnahme in einem ganzen Schutzkonzept sein können, das neben dem konkreten Tätigkeitsbezug die gesamten Abläufe während einer Arbeitsschicht berücksichtigt. In zahlreichen Studien wurden die Zusammenhänge des Infektionsgeschehens unter HCWs mit weiteren Schutzmaßnahmen untersucht. Cattelan et al. [19] bestätigten, dass eine integrierte Strategie der räumlichen Trennung von infektiösen Patient*innen, der konsequenten Anwendung persönlicher Schutzausrüstung, die Überwachung von Temperatur und Symptomen bei HCWs sowie regelmäßige Testungen vor jedem Dienst geeignet sind, Infektionen auch in Hochinzidenzgebieten unter HCWs zu verhindern.

In ihrer Untersuchung unter 212 HCWs im Frühjahr 2020 mittels Antikörperbestimmungen fanden Bahrs et al. [20] keinen Beleg dafür, dass das berufliche Risiko für HCWs größer ist als das private. Sie beobachteten sogar, dass unter den HCWs mit hohem beruflichem Risiko die Infektionsraten niedriger waren als bei HCWs mit mittlerem Risiko sowie bei Beschäftigten aus den Verwaltungsbereichen und begründeten den Effekt mit einer hohen Akzeptanz der persönlichen Schutzmaßnahmen. Eine bis September 2020 durchgeführte Untersuchung mittels Antikörperbestimmungen unter 907 HCWs durch Hildebrandt et al. [21] kam zu einem vergleichbaren Ergebnis. Beide Untersuchungen fanden zwischen der ersten und zweiten COVID-19-Welle statt, allerdings in Zeiten mit niedriger Inzidenz (Sommerplateau). Aber auch eine durch Dusefante et al. [22] von September 2020 bis Januar 2021 durchgeführte Untersuchung unter Berücksichtigung sozialer, persönlicher und beruflicher Variablen in der zweiten Welle kam zu keinem anderen Ergebnis. Die Autor*innen bestätigen einerseits ein großes Wissen sowie eine hohe Akzeptanz unter den HCWs bezüglich Infektionsschutzmaßnahmen und -kontrollen sowie keinen Einfluss der berücksichtigten Variablen [22]. Für die von Hupf et al. [23] seit Pandemiebeginn bis Februar 2021 untersuchten HCWs in der unmittelbaren Versorgung von COVID-Patient*innen konnte unter Einhaltung der Hygiene- und Schutzmaßnahmen ebenfalls lediglich ein niedriges und mit der Gesamtbevölkerung vergleichbares Risiko ermittelt werden. Auch in den beiden letztgenannten Studien wurden serologische Untersuchungen durchgeführt. In ihrer Untersuchung mittels umfassender SARS-CoV-2-PCR-Tests unter mehr als 1000 Beschäftigten bestätigten Schöppenthau et al. [24] deren Schlüsselrolle zur Früherkennung von Infektionen unter HCWs und zur Verhinderung einer weiteren Ausbreitung. Testungen gehören nach den Autor*innen zusammen mit dem konsequenten Einsatz von Schutzmasken für Personal und Patient*innen zu den wirksamsten Schutzmaßnahmen.

Corona-Schutzimpfungen bieten einen wirksamen individuellen Schutz insbesondere vor schweren COVID-19-Erkrankungsverläufen [25]. Allerdings gibt es inzwischen bereits zahlreiche Studien zu Infektionen auch nach stattgehabter vollständiger Immunisierung [26, 27]. Im Rahmen der betrieblichen Hygiene- und Schutzkonzepte sind COVID-19-Schutzimpfungen neben Schutzkleidung und Atemschutzmasken Teil der individuellen Schutzmaßnahmen. Sie ersetzen weder persönliche Schutzausrüstung noch weitere technische oder organisatorische Schutzmaßnahmen. Nach der Einführung der einrichtungsbezogenen Impfpflicht werden geimpfte Beschäftigte in Einrichtungen nach IfSG danach auch nicht von SARS-CoV-2-Testungen ausgeschlossen. In der Nationalen Teststrategie wird nicht nach dem Impfstatus unterschieden [28].

### Anlässe bzw. Ziele für Beschäftigtentestungen

Je nach Schutzziel kommen unterschiedliche Testmethoden in Betracht. Sensitivität, Spezifität der Testmethoden unter Beachtung der Vortestwahrscheinlichkeit bestimmen den prädiktiven Wert zur Einschätzung der Aussagekraft der Methode [18, 29]. Neben der Spezifität und der Sensitivität eines Tests bestimmt auch die Qualität der Probenentnahme selbst die Validität des Testergebnisses. Bei der Auswahl des Testverfahrens müssen alle Einflussgrößen Berücksichtigung finden, insbesondere, wenn kommerzielle Antigen-Tests im Selbsttestungsverfahren im betrieblichen Kontext Anwendung finden sollen. Zuvor müssen jedoch Anlässe bzw. Ziele für die Beschäftigtentestungen geklärt sein.

Dem Arbeitsschutzgedanken folgend wäre eine Ableitung von „Institutional-Tests“ denkbar. Anlässe dafür lassen sich mit den entsprechenden Gefährdungsbeurteilungen für Beschäftigte begründen. Sie stellen eine durch den AG abgeleitete Arbeitsschutzmaßnahme dar. Das Ziel ist die Sicherstellung des Gesundheitsschutzes der Beschäftigten, wenn entsprechende gefahrgeneigte Tätigkeiten („medizinische Versorgung von COVID-19-Patient*innen“) ausgeführt werden. Die Kosten für derartige Tests gingen zu Lasten des AG. Die Einbeziehung der Betriebsärzt*innen wäre sinnvoll. Der Datenschutz wäre sichergestellt.

Testanlässe, die vordergründig den Schutz besonders vulnerabler Patient*innengruppen fokussieren (*Coming-Tests*), sind über die Arbeitsschutzgesetzgebung nicht explizit erfasst, weshalb hier betriebsinterne Regelungen, wie Dienstanweisungen oder -vereinbarungen getroffen werden müssen, um Pflichten zu definieren und Datenschutzregelungen festzulegen. Untersuchungen sind dann zulässig, wenn der Anlass konkret begründbar und mit klarem Bezug zur beruflichen Tätigkeit und der Untersuchungsumfang angemessen und zumutbar ist [30]. Zum Ausschluss von Übertragung von SARS-CoV-2-Infektionen auf besonders zu schützende Patient*innengruppen kann dieser Eignungsaspekt durchaus überlegt und entsprechend angepasst umgesetzt werden.

Tests nach dem IfSG folgen der Überlegung, dass während einer Epidemie oder Pandemie eine hohe Infektionslast in der Bevölkerung besteht. Über Verordnungen bzw. Verpflichtungen für AG, solche „public tests“ in Betrieben anzubieten, sollen breite Bevölkerungsschichten gezielten Untersuchungen zugeführt werden. Im Sinne eines „Monitorings der berufstätigen Bevölkerung hinsichtlich ihres individuellen Lebensrisikos im Setting Betrieb“ müssen allerdings andere Kostenträger als die Unternehmen selbst gefunden werden. Diese Überlegungen treffen ebenfalls auf das Thema Kontaktpersonennachverfolgungen zu. Hier muss rechtssicher unterschieden werden, ob diese nach tätigkeitsbedingtem Kontakt zu einem Indexpatient*innen (berufliche Tätigkeit, (mehrere) Beschäftigte stecken sich an einem infektiösen Patient*innen an) oder im beruflichen Umfeld zu Kolleg*innen (Indexfall Beschäftigte*r, kein eindeutiger Bezug zur Tätigkeit an COVID-Patient*innen) erfolgte.

Nicht zuletzt können Testangebote dazu beitragen, dem individuell wahrgenommenen Risiko einer Ansteckung im Rahmen der Tätigkeit zu begegnen und damit empfundene psychische Belastungen der Beschäftigten zu senken [31]. *Leaving-Tests* könnten auch dann noch angeboten werden, wenn andere Testanlässe entfallen.

**Figure 5:**
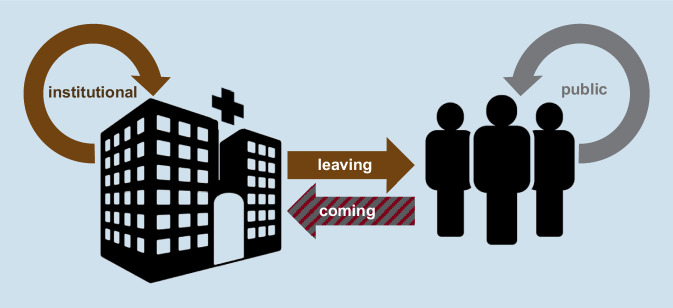

In Abb. 5 sind die Zielrichtungen zu den beschriebenen Testanlässen schematisch dargestellt.

Die durch Schneider et al. [32] untersuchten Ausbrüche von SARS-CoV-2-Infektionen in verschiedenen Bereichen der Patient*innenversorgung bestätigten mehrere ungeschützte Kontakte unter infizierten HCWs sowie im Ergebnis die Notwendigkeit, auch das Übertragungsrisiko zwischen HCWs in Schutzkonzepten zu berücksichtigen. Blain et al. [33] bestätigen aufgrund ihrer Untersuchungen, im Ausbruchsfall alle Kontaktpersonen eines positiven HCW-Indexfalls regelmäßig zu testen, bis eine Ansteckung sicher ausgeschlossen ist.

Hanrath et al. [34] konnten in ihrer Studie in England außerdem nachweisen, dass die Positivrate unter Beschäftigten mit einem Black, Asian oder Minority Ethnic (BAME) oder sozial schwachen Hintergrund signifikant erhöht war. Das betraf beispielsweise Beschäftigte mit Supporttätigkeiten. Die Ergebnisse lassen den Schluss zu, dass die Umsetzung von Schutzkonzepten alle Beschäftigten gleichermaßen erreichen und überzeugen müssen.

## Schlussfolgerungen

Die Umsetzung gesetzlicher Vorgaben in regelkonforme und rechtssichere SARS-CoV-2-Testkonzepte/-prozesse in Einrichtungen des Gesundheitswesens setzt bereits eine integrative und vollzugstaugliche Formulierung von Gesetzen und Verordnungen voraus. Insbesondere die Klärung der Frage, welches Testkonzept welches konkrete Ziel verfolgt, bestimmt nachfolgend den Umgang mit erhobenen Daten, Meldewege, aber auch Zuständigkeiten und ggf. sogar unterschiedliche Kostenträger. Bereits auf der Ebene der Gesetzgebung ist die Einbeziehung sowohl weiterer Ministerien als auch von Fachschaftsvertretungen, Berufsverbänden, Arbeitgeber- und Arbeitnehmer*innenvertretungen, Datenschutzexpert*innen, Vertretungen möglicher Kostenträger (UVT, GKV) unbedingt zu empfehlen. Für settingbezogene Maßnahmen müssen im Betrieb alle Aspekte des Beschäftigtendatenschutzes mitgedacht werden. Das gilt insbesondere für Meldewege nach IfSG, für die IT-gestützte Schnittstellenlösungen für erhobene Beschäftigtenbefunde nicht nur für Labore, sondern bspw. auch für betriebsärztliche Dienste geschaffen werden müssen. Betriebsärzt*innen kennen die Datenschutzanforderungen für den Umgang mit Beschäftigtendaten im Rahmen des Gesundheitsschutzes. Ihre Interessenvertretungen sollten stärker in politische Entscheidungen einbezogen werden.

Die eigentliche Arbeit zur Übersetzung gesetzlicher Anforderungen beginnt aber an der Einrichtungstür. Hier sind AG gefordert, den Beschäftigtendatenschutz sicherzustellen *und* gesetzlichen Meldevorgaben zu folgen, Drittschutz sicherzustellen *und* Kosteneffizienz im Blick zu haben, epidemiologische Daten zu kennen *und* gesetzliche Vorgaben zu erfüllen, Schweigepflichten einzuhalten *und* den Bevölkerungsschutz sicherzustellen. Die wesentlichen Erkenntnisse dieser Forschungsarbeit bestehen darin, dass auf einer betrieblichen Ebene ebenfalls entsprechende Interessenvertretungen (*Krisenstab*) in einem konstruktiven und strukturierten Austausch treten müssen, um unter Beachtung aller Einflussgrößen und Ziele von SARS-Cov-2-Testungen Zuständigkeiten, Ressourcen und entsprechende Unterstützungsprozesse festzulegen. Die klare Trennung, SARS-CoV-2-Testungen als Arbeitsschutzmaßnahme zum Beschäftigtenschutz anzubieten oder sie zum Schutz Dritter (Patient*innen- bzw. Bevölkerungsschutz) verpflichtend einzuführen, bestimmt maßgeblich weitere Prozessschritte. Für Arbeits- und Gesundheitsschutzmaßnahmen gilt die Beachtung der gesetzlich geregelten Schweige- und Datenschutzregelungen. Werden für bestimmte Beschäftigtengruppen verpflichtende Tests vereinbart, müssen ggf. entsprechende Dienstvereinbarungen unter Einbeziehung der Personal- oder Betriebsräte getroffen werden. Entsprechende Lösungen sollten im Sinne von niederschwelligen Testangeboten oder -pflichten für Beschäftigte zentral oder dezentral bereitgestellt werden. Integrative innerbetriebliche IT-Lösungen unterstützen maßgeblich diese Prozesse. Es muss in der Zukunft ein zentrales Anliegen der Betriebe sein, Schnittstellenlösungen für eine datenschutzkonforme Informationsweitergabe zu Beschäftigten zu finden.

Innerbetriebliche Testkonzepte sollten epidemiologische und biostatistische Einflussgrößen berücksichtigen. Die Auswahl der Testmethoden sowie die Häufigkeit durchgeführter Tests müssen einerseits der Inzidenz in der Bevölkerung folgen, andererseits aber auch bestehende Arbeits- und Hygieneschutzkonzepte im Betrieb würdigen. Hier müssen Betriebe aufgrund der zur Verfügung stehenden Expertise autorisiert sein, situationsadaptierte Konzepte zu entwickeln. Pauschale Vorgaben, insbesondere auch zur Abrechenbarkeit von Leistungen konterkarieren das Bestreben, mit bester fachlicher Expertise bedarfs- und zielgerechte Lösungen zu finden. Das schließt die Empfehlung ein, UVT stärker in die Abstimmung zu Arbeitsschutzkonzepten sowie deren Finanzierung einzubeziehen.

Für zusätzlich anfallende Aufgaben müssen entsprechende, auch personelle Ressourcen bereitgestellt werden. Durchführung von PCR-Abstrichuntersuchungen, zusätzliche Laboruntersuchungen, Dokumentation, Meldung und Beratungen von Beschäftigten, Informationsbereitstellung und IT-Schnittstellenkonfigurationen – die Bewältigung dieser zusätzlichen Aufgaben während einer Pandemie gelingt auf Betriebsebene nur über eine interdisziplinäre Zusammenarbeit und ggf. nur, wenn reguläre Aufgaben für einen definierten Zeitraum zurückgestellt oder zusätzliche Ressourcen gegenfinanziert werden. Für solche Ausnahmeszenarien kann die Unterstützung durch die UVT überlegt werden.

Es muss davon ausgegangen werden, dass insbesondere Betriebsärzt*innen in Einrichtungen des Gesundheitswesens über einen langen Zeitraum alle weiteren gesetzlich geforderten Aufgaben zugunsten dringlicher Aufgaben im Rahmen der Pandemiebewältigung^2^ temporär aussetzen mussten. Gesetzgeber und UVT sind gefordert, Überlegungen anzustellen, wie in solchen Zeiten im Sinne einer Priorisierung betriebsärztlicher Aufgaben nach dem ASiG eine Konzentration auf aktuell anfallende Bedarfe gelingen kann. Konkret könnte die Überlegung angestellt werden, z. B. Angebotsuntersuchungen durch die Stärkung einer Wunschvorsorge zu ersetzen.

Die Bewältigung der SARS-CoV-2-Pandemie ist zweifelsfrei eine gesamtgesellschaftliche Aufgabe. Für Maßnahmen in betrieblichen Kontexten müssen zentrale Aspekte wie Sicherstellung des Beschäftigtendatenschutzes oder Bereitstellung von Ressourcen zusätzlich berücksichtigt werden. Mit der vorliegenden Arbeit werden auf Grundlage der in einer Fokusgruppendiskussion zusammengetragenen hemmenden und fördernden Aspekte für die Ableitung insbesondere von SARS-CoV-2-Testkonzepten maßgeblichen Handlungsaufträge für politische und betriebliche Entscheider abgeleitet.

Die Ergebnisse dieser Arbeit werden in einer entsprechenden Handlungsempfehlung zusammengestellt, die als Checkliste für Entscheidung und Planung von Testkonzepten genutzt werden kann.

### Limitationen

In die Ergebnisse sind die Erfahrungen von lediglich neun Teilnehmenden eingeflossen. Insbesondere eine stärkere Beteiligung von Vertreter*innen der gesetzgebenden Seite bzw. deren beratenden Gremien sowie des Öffentlichen Gesundheitsdienstes wäre wünschenswert gewesen. Aufgrund der trotz Nachfragen geringen Beteiligungsbereitschaft wurde nur eine Fokusgruppendiskussion geführt. Es kann daher nicht ausgeschlossen werden, dass nicht alle relevanten Aspekte Berücksichtigung fanden. Auf der anderen Seite konnten insbesondere Vertreter*innen gewonnen werden, die aufgrund ihrer Tätigkeiten in betriebliche Schutzkonzepte für Beschäftigte einbezogen sind und für die Beantwortung der Fragestellung wichtige Impulse geben konnten. Dennoch sollten die gewonnenen Erkenntnisse auch empirisch praxisbezogen untersucht werden, zumal die Vielfalt der unterschiedlichen Einrichtungen des Gesundheitswesens mit möglicherweise differierenden praktischen Voraussetzungen mit der Fokusgruppe nicht abgebildet werden konnte.

Fragen nach möglichen Kostenträgern für unterschiedliche Testkonzepte wurden nicht explizit gestellt und in der vorliegenden Studie zunächst auch nicht weiterverfolgt.

## Footnotes

1 Bundesministerium für Arbeit und Soziales, Bundesministerium für Gesundheit, Bundesanstalt für Arbeitsschutz und Arbeitsmedizin, Deutsche Gesellschaft für Arbeitsmedizin und Umweltmedizin e. V., Der Verband Deutscher Betriebs- und Werksärzte e. V., Deutsche Gesetzliche Unfallversicherung, Verband für Sicherheit, Gesundheit und Umweltschutz bei der Arbeit e. V., Verband Universitätsklinika Deutschland und Gesetzliche Krankenversicherung.

2 Zum Beispiel Unterstützung bei Gefährdungsbeurteilungen; Begleitung des Einsatzes von Schwangeren und Stillenden; Vorschlagen und Begleitung der Umsetzung technischer, organisatorischer und persönlicher Schutzmaßnahmen; Ermöglichung individueller Gesundheitsberatungen; Erarbeitung von Informationen für Beschäftigte; Beantwortung von Eignungsfragestellungen.
